# Energy efficient and sustainable design of a multi-story building based on embodied energy and cost

**DOI:** 10.1038/s41598-024-66769-5

**Published:** 2024-07-13

**Authors:** Zhang Qing Qing, Zhang Li Na

**Affiliations:** Department of Jianghe, Architecture College University, Northeastern University, Shenyang, 110819 China

**Keywords:** Civil engineering, Energy infrastructure

## Abstract

Sustainable multi-story building designs are gaining increasing attention in light of the green development of the building industry. Recently, many studies have been conducted to determine the optimized embodied energy considering size of structural members and materials strength using a single objective function. In this context, the current study adopted a multi-objective function based on cost and Embodied Energy (EE) for the sustainable design of the entire multi-story building. A BuildingEnergy computer program is used to assess the energy consumption performance of a multi-story reinforcement cement concrete building. Based on the proposed method, an analysis is carried out to compare the optimal solutions for multi-story building. Furthermore, a detailed parametric study was conducted to explore the main factors for energy-efficient column and beam design. The results revealed that with a comparison of the most “carbon-friendly” and “cost-friendly” solutions, an added cost of 6–7% can contribute up to a 13% emission reduction. The sectional dimensions, steel rebar, concrete strengths, cost ratio, building height, and eccentricity remarkably influence sustainable design, cost optimization, and minimum carbon emission. Overall, this study could help to define cost-effective and energy-efficient structural members. Eventually, the EE is confirmed to be a feasible parameter for designing more sustainable multi-story RCC buildings.

## Introduction

The construction sector and building accounts for the biggest share in the consumption of natural resources by materials extraction and use of land. Globally, Reinforcement Cement Concrete (RCC) buildings are responsible for between 25 and 40% of total energy use^1,2^. The commercial and residential buildings account for roughly 30% of primary energy consumption and 30% of greenhouse gas emissions in developing countries^3–5^. Energy-efficient and cost-effective building designs are essential to satisfy the rapidly increasing demands for enhanced green economy and sustainability in developing countries^6–8^. Buildings are among the main sectors of energy use, as they are responsible for 39% of global carbon emissions and consume about one-third of global energy usage^9^. A large number of worldwide programs and commitments are in place to improve sustainability and build energy efficiency through their life cycle^10–12^. The energy efficiency of buildings is a vital element that could significantly increase energy security, decrease greenhouse gas emissions, and help economic development in construction industry^13^.

The construction industry and design experts have widely adopted several energy-efficient measures that significantly reduce a building's operational energy consumption^14–16^. Before a building’s use, the manufacturing of building materials have a significant off-site effects. These effects mostly happen in the life-cycle stages of the material acquisition, transport, and distribution. This upstream energy capital is measured in terms of the embodied energy of a particular building materials. It should be noted that the embodied energy includes not only the energy used upstream but also the energy used during on-site construction and the energy required to replace materials and components throughout the useful life of the building^17–19^. In addition, optimizing the embodied energy of any given building is an important task that depends greatly on the size of structural elements, material strength, loads and optimized function. To address this, a "long view" approach that considers the all above parameters, is necessary for sustainable building design based on EE and cost^20,21^.

Nowadays, Embodied Energy (EE) has become an important tool in the sustainable design of buildings because of many reasons. First, the civil engineering field is an energy-demanding with about big contribution to primary energy use worldwide^20,21^. Second, small renewal cycles and the usage of less resilient materials can increase the EE of the buildings^13^. The embodied energy of building materials can make up a sizeable portion of a country's energy consumption. Rough estimates for the UK, USA, Singapore, China, Pakistan India, and Nepal indicate that materials account for 10% of energy use^22–25^. According to the findings of prior studies, the amount of EE of total life-cycle energy can range from 5 to 40%^26^. This wide amount is mostly caused by the fact that embodied energy differs between countries. Additionally, as efforts to make net-zero energy buildings progress, these percentages will rise^27,28^. This is because the net-zero energy goal pertains largely to the operating energy, rather than to the life-cycle energy.

In modern construction, RCC buildings are one of the most popular structural forms, which are considered to be responsible for substantial amounts of cement and steel rebar consumption. In this context, several studies have been carried out to examine the embodied emissions from RCC buildings. Such as, Zhang et al.^29^ performed scenario analysis to investigate the embodied emissions of multi-story residential buildings in China via CO_2_ emission. Sandaruwan et al.^30^ assessed the embodied emissions of green office buildings in Sri Lanka. The outcomes revealed that the composite and concrete buildings produced more emissions than the steel structure. Nie et al.^31^ analyzed CO_2_ emissions from plaza ground and found that the building main elements can contribute more than 70% of the total embodied emissions. Li et al.^32^ compared the carbon footprint in prefabricated buildings. He reported that the selection of load-bearing systems and the number of stories significantly change the low-carbon design scheme. Zhang et al.^33^ compared the embodied emissions of cast-in-place and precast construction methods for RCC buildings. The results showed that the precast technology can reduce the total embodied emissions by improving the construction and production efficiency. The more recent studies demonstrate that RCC buildings can be more carbon intensive than timber and steel buildings, and structural elements are influential factors for building embodied emissions.

With respect to optimized structural elements, both singly reinforced and doubly-reinforced beams were considered^34–36^, yet none of the studies has considered column sections, concrete and steel strength and eccentricity. In addition, few studies have considered two objectives^37–39^, all of them have adopted single-objective optimization algorithms. In detail, only one objective of cost, energy, or minimum emissions was considered at a time, instead of considering multi-objectives simultaneously. A coupled multi-objective structural optimization still needs to be conducted concerning the tradeoff between the cost and emissions. To investigate a part of the knowledge gaps, the present study adopts a multi-objective optimization algorithm, which aims at the sustainable design of RCC multi-story building according to the tradeoff between the costs and EE.

This paper aims to investigate the implications of utilizing carbon emission and EE as the objective function to find out a sustainable building design based on the size of structural elements. A BuildingEnergy computer program is also used to assess the energy consumption performance of a multi-story RCC building. For comparative analysis, the implications of EE are also deeply studied for a case in which the total cost is used as the objective function. For each case, the role of rebar cost ratio to that of concrete is also established in this study.

### Reference building

**Figure 1:**
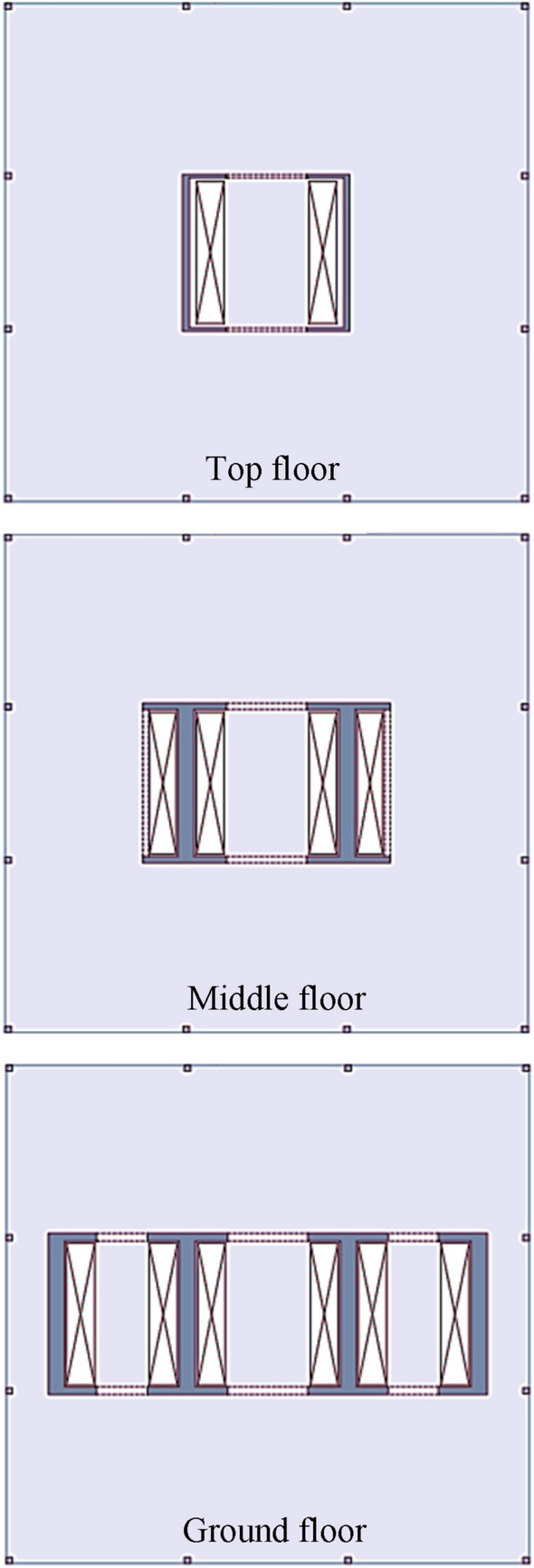
Plan of the central core and columns of the building.

Construction of multi-story buildings is frequently designed to be energy efficient and cost-effective; in contrast, a small amount of research has been conducted on the embodied energy of structure^1,40^. A 50-story multi-story building composed of Reinforcement Cement Concrete (RCC) is taken as example. The aerial view of the central core at different heights and the columns of the building are shown in Fig. 1. Three horizontal sections of the core and the position of columns are shown in (Fig. 1). In the reference multi-story building, columns are placed only at the perimeter of the building (Fig. 1). Furthermore, Fig. 1 shows the reduction of core section, from the bottom to the top of the structure, which further increases the free spaces of the floors.

**Figure 2:**
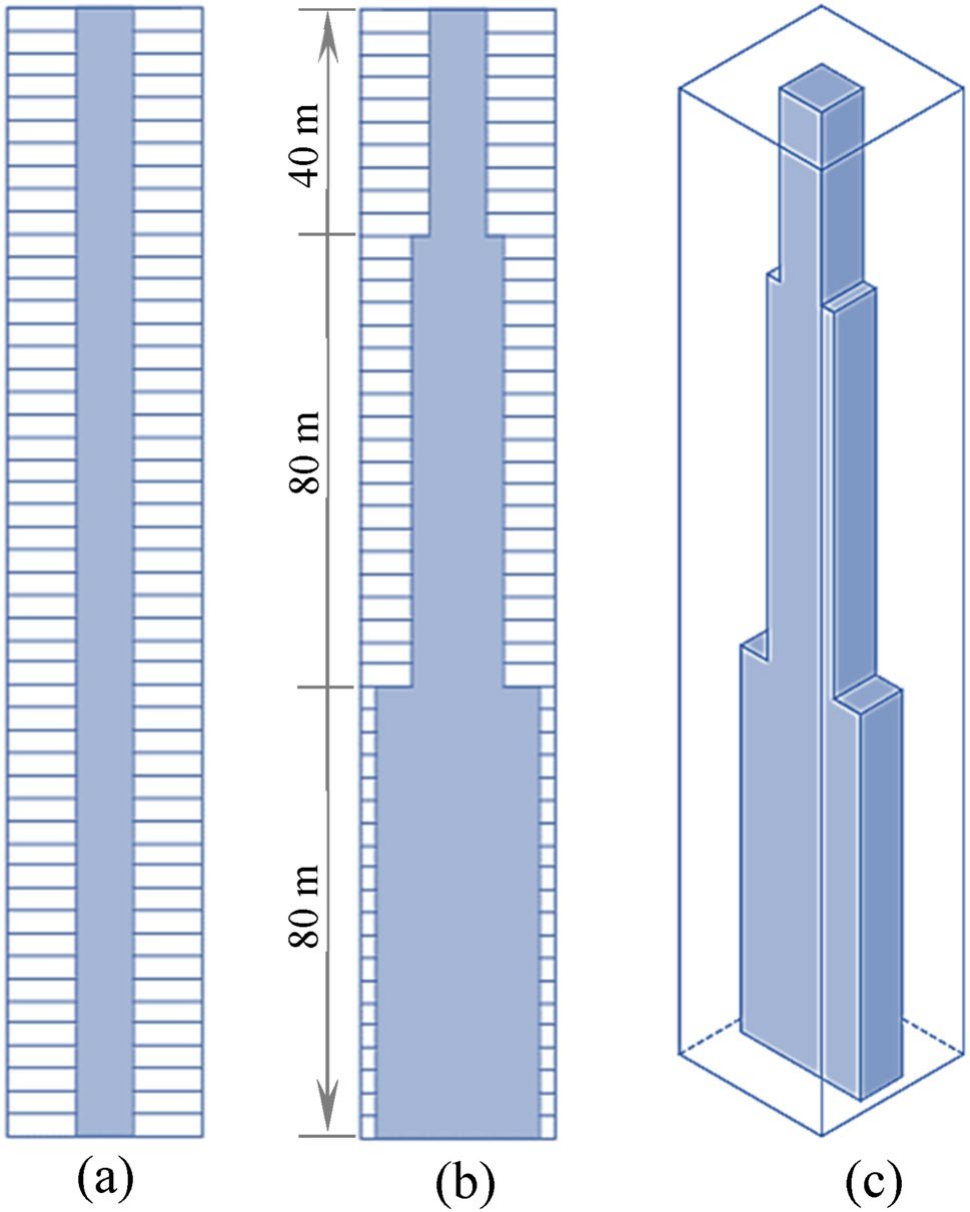
(**a**) Central core in elevation, (**b**) vertical section, and (**c**) 3-D shape of the core; tapering from the bottom to the top of the building.

The building has an EE per square meter of gross floor area of 19 GJ/m^2^. The inter-floor height of the building is 4 m. The height of each floor was kept the same to attain consistent and more accurate results. The columns and beams of the building are positioned at the boundary of the building as shown in Fig. 1. The elevator shafts, services, and stairs have an approximate area of 20% of the total gross floor area of each floor. This percentage varies due to the tapering of the core at the 20th and 40th stories (Fig. 2). The reference building considered in the analyses is composed of a central core made of reinforcement cement concrete (Fig. 2). As a result, the core contributes importantly to the lateral capacity of the building^41^. The tapering in elevation and the shape of the core are based on research of existing multi-story buildings^34,42^.

## Methodology

### Problem statement

Take an RCC rectangular beam and column having a cross-sectional area of *b* x *h* and a length of *L* (7000 mm), where *h* and *b* are the height and width of the beam, respectively. The beam is supposed to have a bending moment of *M*_*u*_ = 500 kN-m and the shear force of *V*_u_ = 250 kN at their critical locations. Due to self-weight, the beam is subjected to a shear force of *V*_*sw*_ and bending of *M*_*sw*_. The beam design, including the standard design of the shear rebar and longitudinal rebar, is according to the guidelines and the restrictions found in the manual of the Chinese design code GB 50010^43^.

Ultimate strength design (USD) is a feasible method, in which *V*_*n*_ ≥ *Ṽ*_*u*_/*ϕ*_*s*_ and *M*_*n*_ ≥ *Ṁ*_*u*_/*ϕ*_*b*_; where *Ṽ*_*u*_ = *V*_*sw*_ + *V*_u_ and *Ṁ*_*u*_ = *M*_*u*_ + *M*_*sw*_, *V*_*n*_ is the nominal shear strengths and *M*_*n*_ is the nominal bending moment, is applied to define the strength of material. *ϕ*_s_ and *ϕ*_*b*_ and are the equivalent strength reduction constants. Describing a reasonable segment as one that fulfills all the requirements of the Chinese design code GB 50010^43^, the objectives of this research are to determine a feasible design of a building that reduces the total embodied energy (EE) and the total cost.

### Design variables

**Figure 3:**
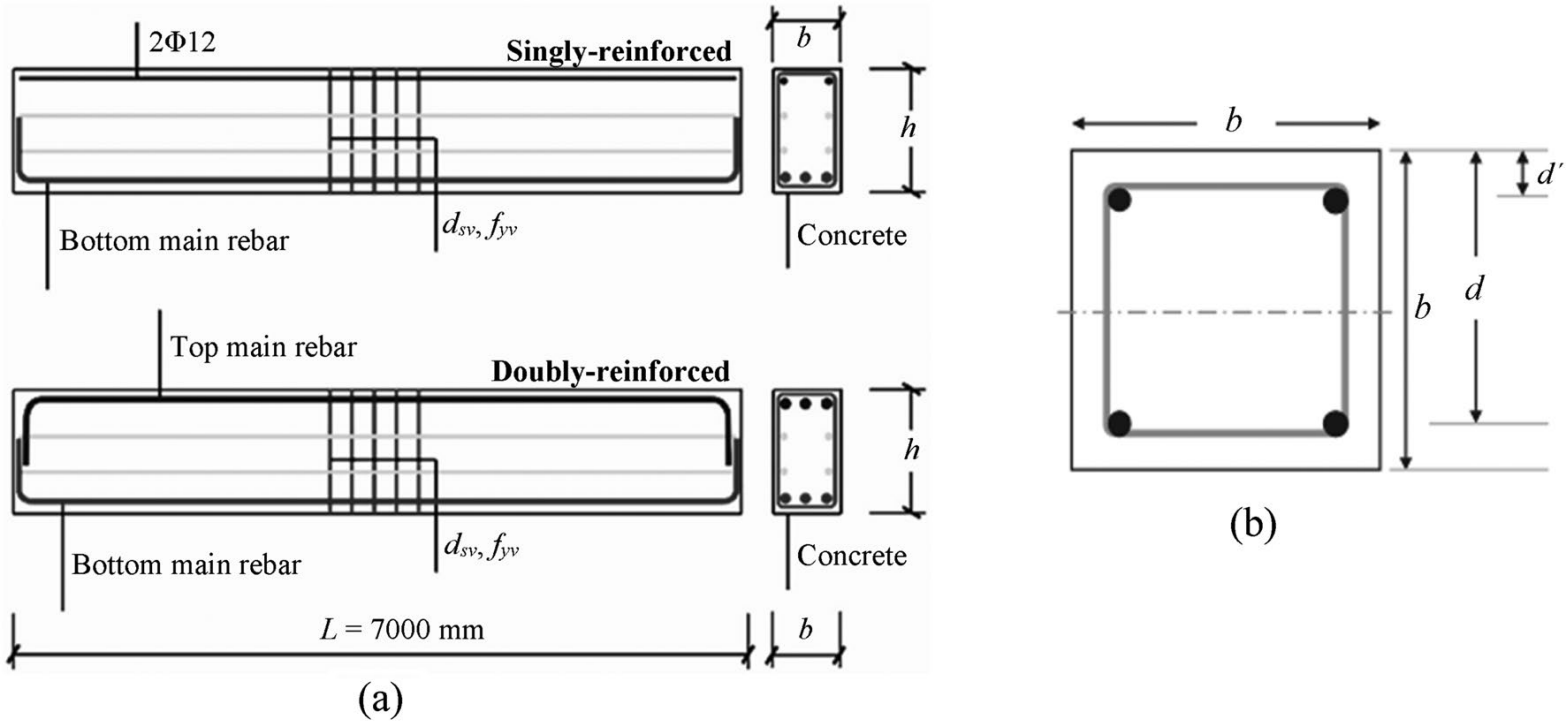
Design variables of (**a**) singly and doubly reinforced beam and (**b**) RCC column.

Design variables are theoretically uninterrupted variables for optimization and better results may be attained consequently by selecting the appropriate value of these variables^44^. In this study, the design variables are beam height (*h*), beam width (*b*), the area of shear rebar (*A*_*v*_), area of longitudinal rebar (*A*_*s*_) (Fig. 3a). The spacing (s) of longitudinal rebar is 200 mm. Two types of beam, one is singly reinforced (SR) beam and doubly reinforced (DR) were chosen as shown in Fig. 3a. The beam length was assumed as *L* = 7000 mm. RCC column with a square section (*b*^2^), subjected to moment and axial force, is also considered as design variable. RCC column consists of four vertical rebar, shear stirrups and concrete and concrete cover *d*´ (Fig. 3b). The failure characteristics of the column were determined by the geometric composition of the column section as well as the eccentricity (ratio of axial force and moment). All design variables are according to the Chinese design code GB 50010^43^ as listed in Table 1. The area of main rebar (*A*_*s*_) and the area of stirrups (*A*_*v*_) are considered continuous variables (Table 1). For specific RCC buildings, the cold environment was specified by the Chinese design code GB 50010^43^.

**Table 1:** Design variables and corresponding ranges of beam sections, from^43^.

Variable	Range
*A*_*s*_	As per GB 50010
*A*_*v*_	As per GB 50010
*b*	200 mm ≤ b ≤ 500 mm
*h*	300 mm ≤ h ≤ 800 mm

### Material strength parameters

The material strength parameters, defined as constants during the optimization process in this study, are listed in Table 2. The strength reduction factor for factor moment (*M*_*u*_) is determined by the net tensile strain of the bottom longitudinal rebar. The value of the concrete constant (λ) is kept 1, which is according to the specific mass of concrete (*ρ*_*c*_) as shown in Table 2. The tensile strain of the longitudinal rebar is selected to define the appropriate value of the strength reduction constant (*ϕ*_*b*_) for bending. The minimum value of *ϕ*_*b*_ is kept 0.65 at the compression strain of concrete *ε*_*cu*_ = 0.003, while the tensile strain of the steel rebar *ε*_*t*_ = 0.002. When *ε*_*cu*_ = 0.003 and *ε*_*t*_ = 0.005, the maximum value of *ϕ*_*b*_ = 0.90 is chosen for analysis at the yielding of main steel rebar. The strength reduction factor was estimated through direct interpolation for intermediary values of the strain. The tensile strain was more than 0.004 for a beam, which parallels *ϕ*_*b*_ = 0.812 (Table 2). These requirements limit the maximum steel rebar in the beam. It must be noted that the spacing of stirrups and the number of main steel rebar are determined according to the Chinese design code GB 50010^43^. Furthermore, other design variables, such as the anchorage lengths of rebar and the number of stirrup legs, were calculated according to the Chinese design code GB 50010^43^.

**Table 2:** Design variables and corresponding values.

Variable	Value
Factored moment	*M*_*u*_ = 500 kN.m
Factored shear force	*V*_*u*_ = 250 kN
Concrete compressive strength	*f*_*c*_ = 34 MPa
Longitudinal reinforcement yield strength	*f*_*y*_ = 420 MPa
Shear reinforcement yield strength	*f*_*yt*_ = 300 MPa
Modulus of elasticity of steel	*E* = 2 × 10^5^ MPa
A specific mass of concrete	*ρ*_*c*_ = 2400 kg/m^3^
A specific mass of steel	*ρ*_s_ = 7850 kg/m^3^
Lightweight concrete factor	λ = 1
Strength reduction factor for shear	*ϕ*_*s*_ = 0.75
Strength reduction factor for bending	0.8 ≤ *ϕ*_*b*_ ≤ 0.9
The ratio between the depth of the stress block and the neutral axis depth	*β*_1_ = 0.8
Maximum useable compression strain in the concrete	*ε*_*cu*_ = 0.03
Section length	*L* = 8 m
Concrete cover for beam and column	*d´* = 70 mm
Longitudinal spacing of shear reinforcement	*s* = 200 mm
Eccentricity	8–460 mm

### Objective functions

Generally, the sustainable and energy-efficient design of RCC buildings refers to the costs and emissions of embodied energy that could be expressed as an objective function^28^. Objective function (*g*) relates to the total embodied energy (EE) per unit length, while objective function (*f*) relates to the total cost of the beam per unit length. The objective functions for total EE and total cost are given below in Eqs. (1) and (2).

1 $$g\left(b,h,{A}_{s}, {A}_{v}\right)= {\rho }_{s}\left({A}_{s}+\frac{{A}_{v}}{s}\right){E}^{s}+L\left(bh-{A}_{s}-\frac{{A}_{v}}{s}\right){E}^{c}$$

2 $$f\left(b,h,{A}_{s}, {A}_{v}\right)= {0.01RC}^{C}\left[{\rho }_{s}\left({A}_{s}+\frac{{A}_{v}}{s}\right)+(bh-{A}_{s}-\frac{{A}_{v}}{S}\right]$$

In Eq. (1), *E*^*C*^ shows the total embodied energy per cubic meter of concrete and *E*^*S*^ represents the total embodied energy per kilogram of steel rebar. In Eq. (2), *R* is the ratio of the cost of 100 kg of steel rebar and the cost of concrete per cubic meter, C^C^ is the cost of concrete per cubic meter and *ρ*_*s*_ is the unit mass of rebar.

In this study, *R* is treated as a variable, while C^C^ was fixed at CNY150/m^3^. Three representative values of *R* were collected from the previous research as presented in Table 3. The variation in *R* is because of the installation costs of steel rebar, placement costs of concrete, and the demand and supply-driven fluctuations in the prices of concrete and steel from year to year are included here. The embodied energy (EE) values in this research are *E*^*S*^ = 8.9 MJ/kg and *E*^*C*^ = 3180 MJ/m^3^, for recycled reinforcement *f*_*y*_ = 420 MPa and for concrete *f*_*c*_ = 34 MPa, respectively^45^. The cost of other constructional materials is taken from previous study^46^.

**Table 3:** Cost ratio variation.

Reference	*R*	Comments
Paya-Zaforteza et al.^55^	1.12	*f*_*c*_ = 35 MPa, *f*_*y*_ = 400 MPa material costs (2007 prices)
Sahab et al.^56^	0.92	*f*_*c*_ = 35 MPa, *f*_*y*_ = 460 MPa, material costs (2001 prices)
Guerra et al.^57^	0.82	*f*_*c*_ = 28 MPa, *f*_*y*_ = 420 MPa material costs (2018 prices)

### Design constraints

According the Chinese design code GB 50010^43^, the safety of RCC buildings subjected to the combined or individual actions of torsion, axial force, shearing, and bending can be ensured by:

3 $${G}_{safe}\left(X, Y\right)={(R}_{d}/{y}_{o}{S}_{d})-1\ge 0$$

In Eq. (3), *G*_*safe*_(*X*, *Y*) shows the constraint related to safety requirements; *S*_*d*_ is load effect,* R*_*d*_ is designed resistance and *γ*_0_ is an significance constant which equals 1.0 for RCC buildings^43^. The designed resistance of given concrete cross-section could be calculated according to the position of rebar and ductility requirements. The serviceability of RCC components including crack limits and deflection could be defined by:

4 $${G}_{serv}\left(X, Y\right)={1-(D}_{d}/{D}_{lim})\ge 0$$

where *G*_*serv*_(*X*, *Y*) represents the constraint related to serviceability requirements. *D*_*d*_ is crack width of the concrete section estimated according to the values of design variables; *D*_*lim*_ is the limit values quantified by design codes.

Moreover, the solution X will be considered as a reasonable if the design solution X can satisfy all the afore-defined constraints. Otherwise, an indicator of constraint violation (ICV) is applied to evaluate the degree of violating the constraints:

5 $$ICV\left(X, Y\right)= \sum{{l}_{1}}\text{max}[-{G}_{safe,l1}\left(X, Y\right),0]+\sum{{l}_{2}}\text{max}[-{G}_{serv,l2}\left(X, Y\right),0]$$

In Eq. (5), *l*_1_ and *l*_2_ are the numbers of constraints related to the safety and serviceability requirements, respectively. It can be seen that, if *X* is a feasible solution, then *ICV*(*X*, *Y*) equals zero; otherwise, *ICV*(*X*, *Y*) takes a positive number.

### Formulation of the optimization problem

The obvious procedure of the optimization problem is given by Eqs. (1) and (2) and it is subjected to factored moment (*M*_*u*_) as

6 $${M}_{u}={\phi }_{b}{A}_{S}{f}_{s}d\left(1-\frac{{\beta }_{1}}{2}\frac{{\varepsilon }_{cu}}{({\varepsilon }_{t}+{\varepsilon }_{cu})}\right)$$

where *f*_*s*_ = *E*_*εt*_ ≤ *f*_*y*_ is the tensile stress in the steel bar. *d* is the distance from the centroid of the longitudinal tensile steel bar and the extreme compression fiber.

7 $$d=h-d$$

And if the maximum yield strength of the longitudinal rebar is *f*_*y*_ and concrete compressive strength *f*_*c*_ then the structure satisfies the following condition

8 $$max\left(0.25\sqrt{{f}_{c}},1.4\right)\frac{bd}{{f}_{y}}\le {A}_{s}\le 0.85{f}_{c}{\beta }_{1}bd\left(\frac{{\varepsilon }_{cu}}{0.004+{\varepsilon }_{cu}}\right)$$

Factored shear force *V*_*u*_ and area of the shear rebar *A*_*v*_ are given in Eqs. (9) and (10),

9 $${V}_{u}={\phi }_{s}\left(\frac{\lambda \sqrt{{f}_{c}}b\theta }{6}+\frac{{A}_{v}{f}_{yt}\theta }{\check{s}}\right)$$

10 $${A}_{v}\ge max\left(\frac{\sqrt{{f}_{c}}}{16},\frac{1}{3}\right)\frac{b\check{s}}{{f}_{yt}}$$

In Eqs. (9) and (10), *f*_*yt*_, ϴ, and $\check{s}$ are the yield strength of shear reinforcement, the diameter of the shear bar, and spacing, respectively. Spacing (s) is taken as

11 $$300 \,\text{mm}\le b\le 800 \,\text{mm}, 300 \,\text{mm}\le h\le 800 \,\text{mm}$$

In Eqs. (6–10), all limitations concerning the design of RCC beams and columns are according to Chinese design code GB 50010^43^. The design strength of a structural member for bending loads is achieved through Eq. (6), so satisfying both force equilibrium and strain compatibility of reinforcement and concrete. The maximum and minimum tensile steel bar of the beam is shown in Eq. (9). Equation (10) is used to determine the design strength of the member. Equation (11) states the upper and lower bound limits on the section depth *h* and width *b* and the requirement of a minimum amount of shear reinforcement.

### Optimization algorithm

Usually, a set of best optimum solutions can be attained in a multi-objective optimization problem. A model of Pareto front^27,28^ was chosen by taking the two differing objectives within the reasonable space defined in Eq. (12)

12 $$\left\{\begin{array}{c}{Z}_{1}\preccurlyeq {Z}_{2} \\ \forall p\in \left\{1, 2\right\} :{f}_{p}\left({Z}_{1}\right)\le {f}_{p}\left({X}_{2}\right) \\ \forall q\in \left\{1, 2\right\} :{f}_{q}\left({Z}_{1}\right)<{f}_{q}\left({Z}_{2}\right) \left(q\ne p\right)\end{array}\right.$$

In Eq. (12), Z_1_ ≼ Z_2_ shows Z_1_ controls Z_2_. *Z*1 and *Z*_2_ are two feasible solutions; *f*_1_ and *f*_2_ are two objective functions. For all possible realistic solutions, if no alternative solution controls Z_1_, then Z_1_ is a non-dominated solution^27,28^. Based on the above-defined problem, a multi-objective Genetic Algorithm (GA) was implemented in Matlab software to optimize the RCC multi-story building. The main procedure is adopted from Zhang and Zhang^27,28^ study.

After defining the objective function and optimization algorithm, a BuildingEnergy computer program is utilized to simulate the energy performance of the building. BuildingEnergy has been validated using ANSI/ASHRAE Standard 140-2004 (Standard Method of Test for the Evaluation of Building Energy Analysis Computer Programs) in this study^47^. The BuildingEnergy software is assembled with a non-steady-state heat transfer model, in which the outdoor air indoor air, and the building envelope are divided into hundreds of nodes^48^. For each node, the energy conservation equation is based on the implicit difference technique. In the temperature field, all equations for nodes form a suitable matrix first and then the temperature field is simulated by solving the matrix through the Gauss-Seidel iteration model.

## Results

### Feasible design domains

**Figure 4:**
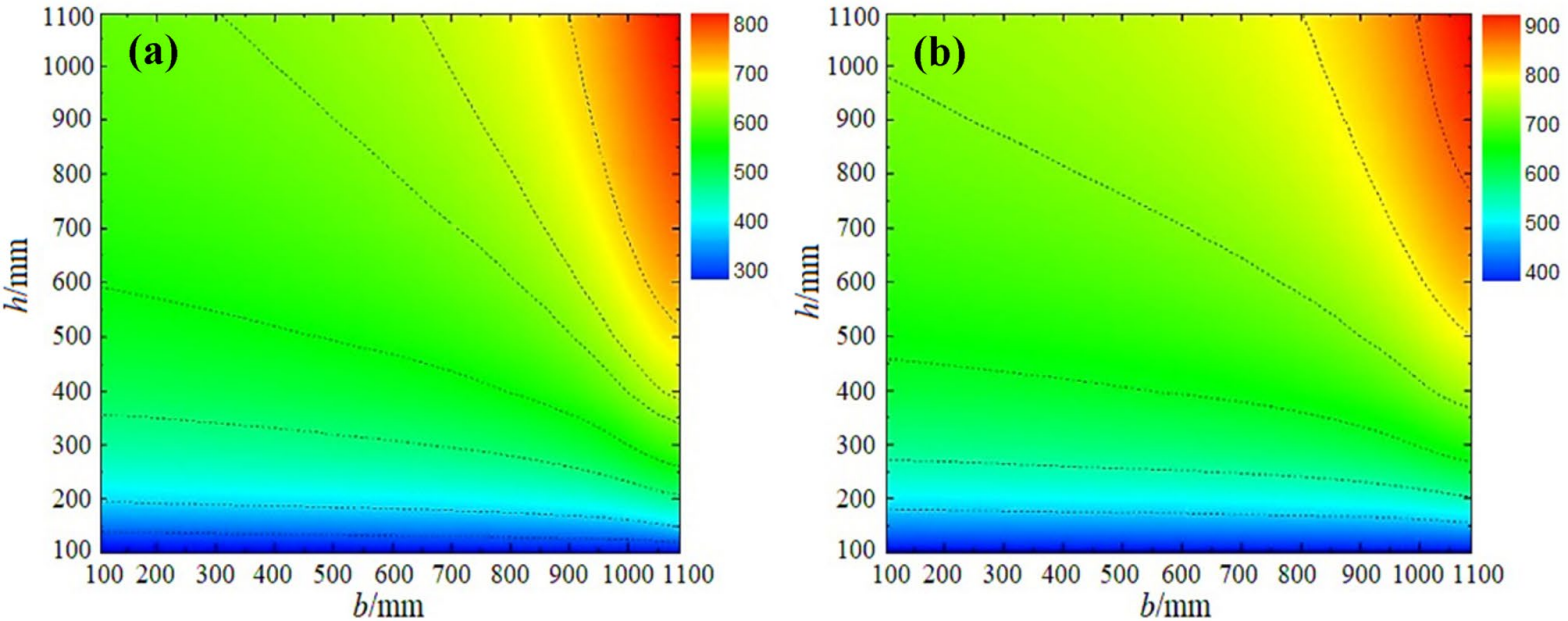
Domain of energy-efficient building designs. (**a**) Total section cost (CNY) and (**b**) total embodied energy (MJ).

In this sub-section, the total cost and embodied energy (EE) are studied using the BuildingEnergy computer program. The climate data used in BuildingEnergy were the typical yearly meteorological data provided by the Chinese Architecture-specific Meteorological Data Sets for Thermal Environment Analysis. Fig. 4 shows the contour maps of total section cost and total section embodied energy at cost ratio *R* = 0.7. The infeasible region shown by the less contour value or blue color region in both maps corresponds to the region where one or more of the constraints are not satisfied. Comparing Fig. 4a and b, it is clear that the lowest cost regions do not necessarily correspond to the lowest embodied energy regions.

**Figure 5:**
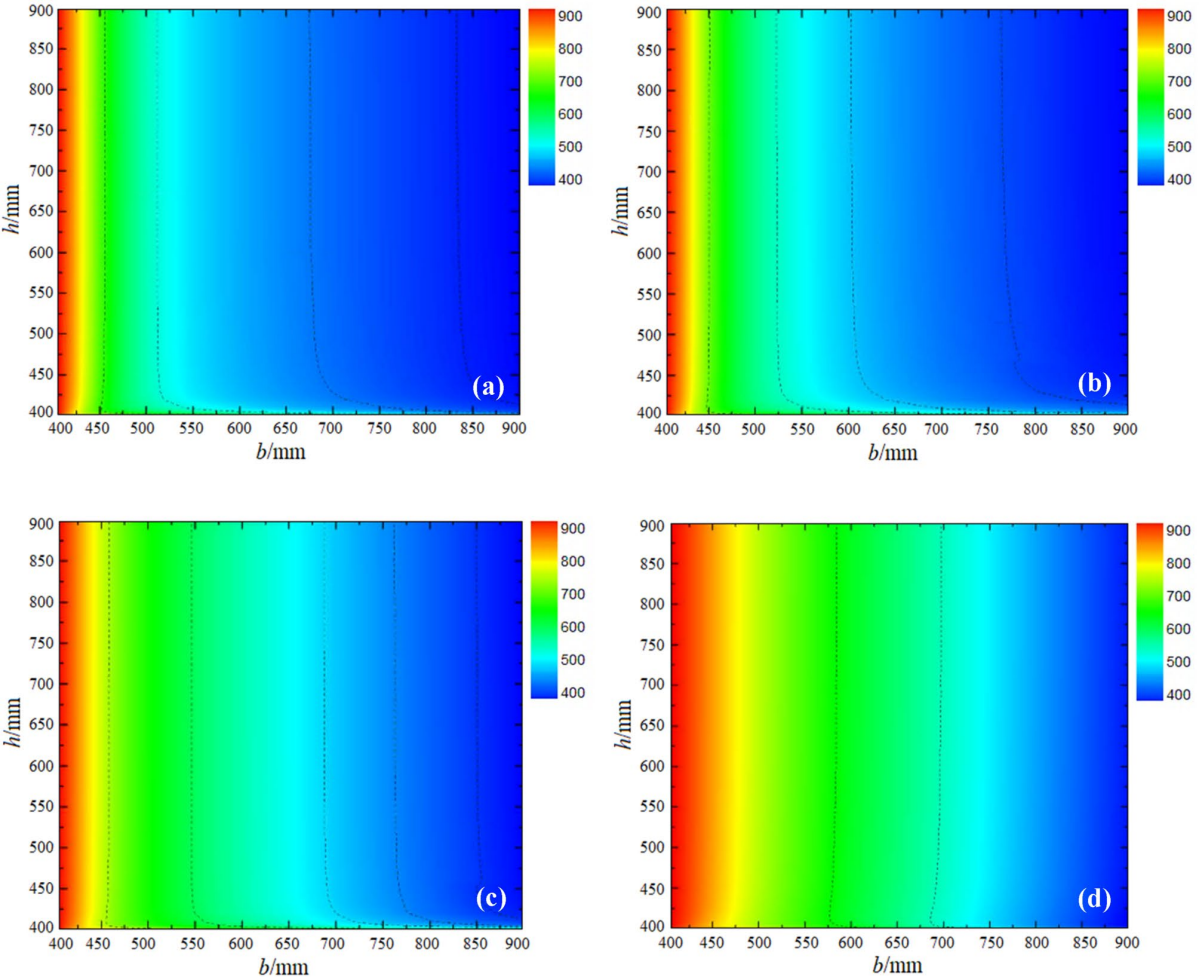
Contributions of concrete and reinforcement to the total cost and total embodied energy. (**a**) Concrete in cost (%), (**b**) rebar in cost (%), (**c**) concrete in embodied energy (%) and (**d**) rebar in embodied energy (%).

It is of great importance to deeply and analyze the dissimilarities in the influences of steel rebar and concrete and their contributions to the total EE versus the total cost. For this purpose, the contour map of the contributions of the steel rebar and concrete to the total cost is presented (Fig. 5a, b). Fig. 5c, d displays a similar contour map for the total EE of both concrete and steel rebar. The blue color in this figure shows less or zero cost as well as embodied energy. It is clear from the comparative analysis that the contribution of steel to the total cost and embodied energy is remarkably smaller than the contribution of concrete.

### Optimized designs

**Figure 6:**
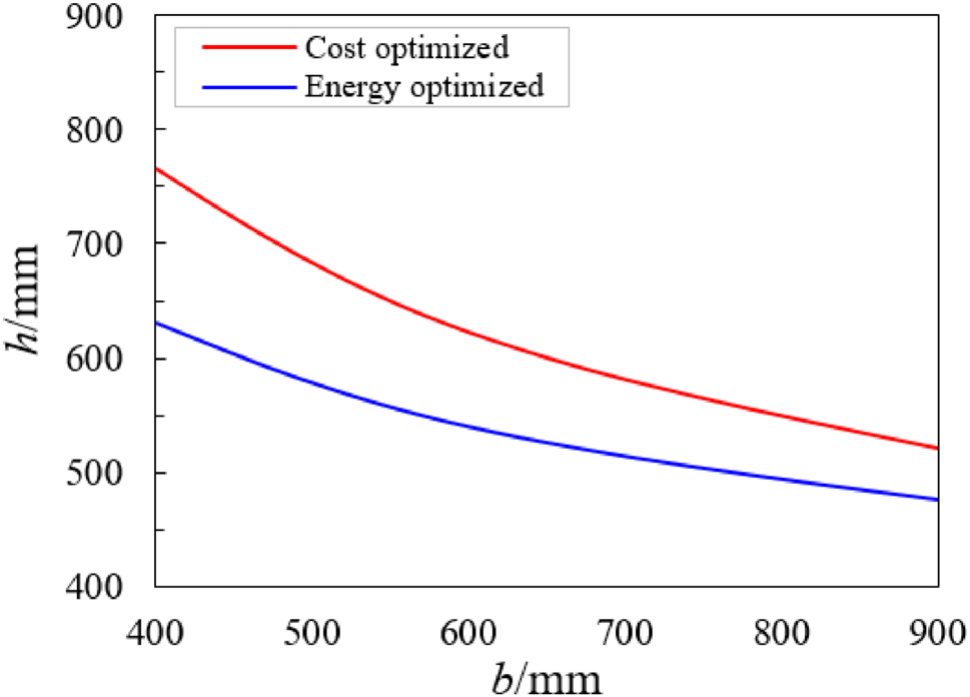
Variation in beam height with beam width for EE and cost-optimized designs.

**Figure 7:**
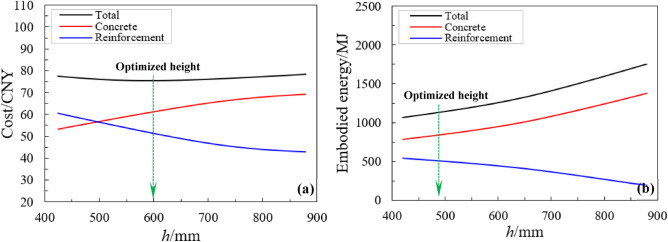
Optimized section design for *b* = 400 mm and *R* = 0.7 (**a**) total section cost and (**b**) total embodied energy.

Consider a 50-storey RCC building, where the cost ratio is *R* = 0.7 and the beam width is 400 mm. Now take the identification of EE and minimum cost to describe an optimal beam size for each value of section width (*b*) ranging from 400 mm to 900 mm as presented in Fig. 6. The embodied energy-optimized sections have lower section heights than the cost-optimized sections, which implies that the former sections use a larger amount of steel rebar and have a smaller concrete volume in comparison to the latter (Fig. 6). The sections of minimum total cost, concrete and steel rebar, and the sections of minimum total embodied energy (EE), concrete and steel rebar, as a function of depth (*h*) are shown in Fig. 7a, b. The overall values of beam height for sections of total cost and minimum embodied energy are marked with a dotted line (Fig. 7a, b). The heights for the optimized sections are 600 mm for total cost and 486 mm for embodied energy as shown in Fig. 7.

Figure 7 illustrates that the minimum section for EE has a larger amount of steel rebar and a smaller volume of concrete in comparison to the minimum cost. The dissimilarities in the amount of steel rebar and section height (*h*) could cause the optimized members to act contrarily. The tensile strain, for cost-optimized sections, of the steel rebar is higher than 0.006 at the maximum concrete compressive strain of 0.004, however it is 0.0053 for the EE-optimized members. Thus, this indicates that the higher ductile behavior of multi-story RCC buildings could be anticipated for cost-optimized members. On the other hand, the ductility for the EE-optimized sections is adequate.

### Parametric study

#### Effect of cost ratio *R*

**Figure 8:**
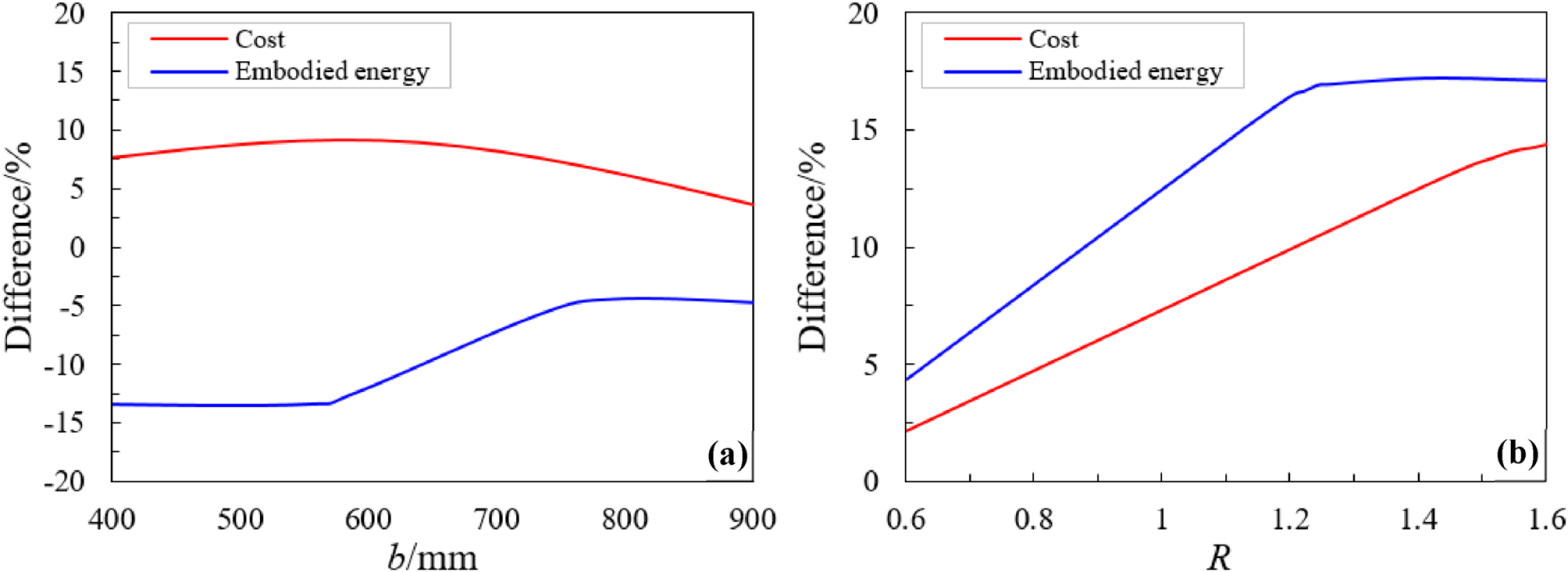
(**a**) Variation in cost and embodied energy with beam width and (**b**) variation in percentage difference in cost and embodied energy with cost ratio *R*.

To investigate the effect of changing values of *R* on the decreasing rate embodied energy is calculated by using the optimization algorithm. The value of *R* is adjusted within the range of 0.6 and 1.6. The variation in embodied energy and the cost of optimized beam width and cost ratio (*R*) is illustrated in Fig. 8a. The difference in both cost and embodied energy between the cost and embodied energy optimized sections is plotted in Fig. 8b. Figure 8a shows that as the cost ratio (*R*) of steel rebars increased from *R* = 0.6 to *R* = 1.6, the embodied energy decreased up to approximately 13%. Simultaneously, within this range, the embodied energy optimized section also experiences an increasing trend in the total cost, amounting to approximately 6%. As the cost ratio *R* goes beyond unity, the differences between the cost additions diminish and the embodied-energy reduced quickly. Fig. 8b shows the difference in the total cost and total embodied energy for both the minimum cost sections and the minimum embodied sections as functions of section width *b*. The results show that optimization for embodied energy can achieve around a 12% decrease in embodied energy at an added cost of roughly 5%.

#### Effect of building height

**Figure 9:**
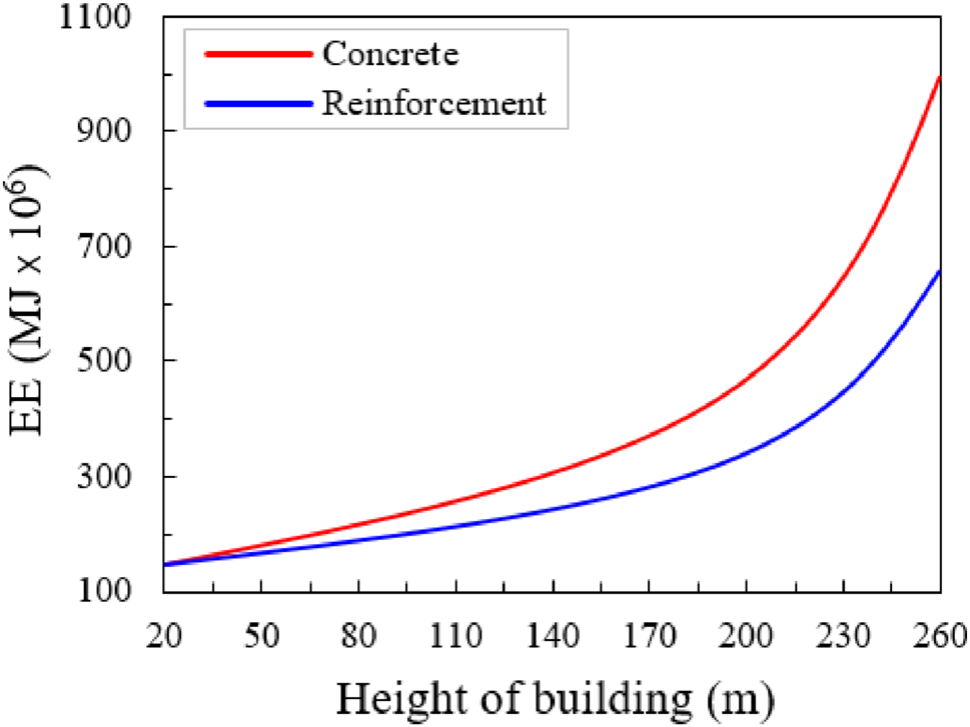
Total EE consumed by the concrete and rebar (reinforcement).

This sub-section analyses the relationships between the building height and embodied energy by using optimized function. The EE values are estimated from the weights of the beams and columns^28^. In the entire building, consumed EE increases significantly as the building height increases as presented in Fig. 9. The concrete elements consume more embodied energy than the steel rebar (Fig. 9). When the building height was between 20 and 170 m the amount of EE increased almost linearly, and after that, it rose rapidly and gave the maximum EE of 1003 × 10^6^ MJ, for concrete, at the height of 260 m (Fig. 9). Figure 9 also plots the relationship between EE consumed by the steel rebars and building height. In the case of steel rebar, the maximum amount of consumed EE was 670 × 10^6^ MJ as can be noted in Fig. 9.

**Figure 10:**
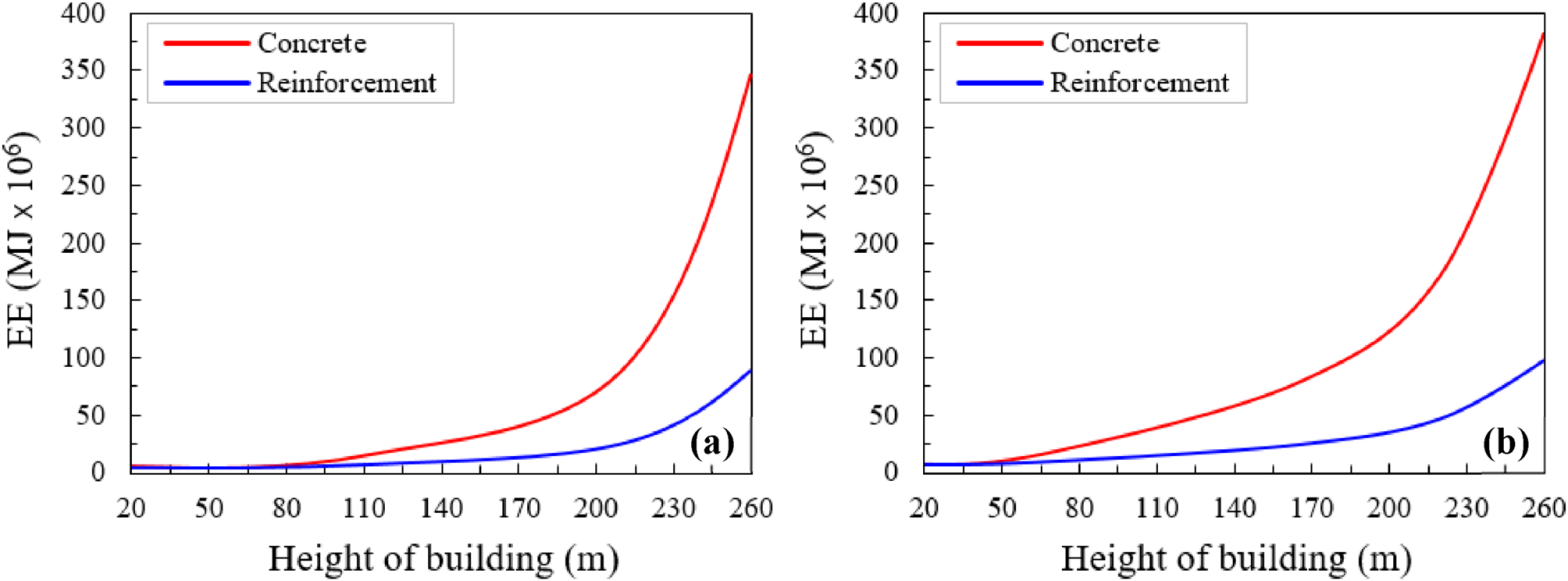
(**a**) Total EE consumed by the columns, as a function of the height of the building and (**b**) total EE consumed by the beams, as a function of the height of the building.

Building height has a remarkable effect on EE. The increasing rate of the total embodied energy concerning building height shows an exponential trend (Fig. 10). Because the EE also depends on building height directly^28^. In addition, the taller the building the greater the size of the elements which consumed more EE^49,50^. Steel rebar consumes less EE as compared to concrete. The relationship between EE, consumed by columns and beams, as a function of building height, is deeply studied in the research for better understanding (Fig. 10). In the case of columns, when the building height was between 20 and 90 m consumed amount of EE does not increase as shown in Fig. 10a. After that, it increases slowly up to a height of 200 m, and after that, it rises rapidly and gives the maximum value of EE of 330 at the height of 260 m (Fig. 10a). At the building height of 260 m, the maximum EE of 85 × 10^6^ MJ is recorded for columns of steel structure (Fig. 10a). Figure 10b shows the maximum amount of EE consumed by beams of reinforcement and concrete building. In the case of beams, EE first slowly rises for a building height of 20–200 m, and after that, it rises sharply, as shown in Fig. 10b. The maximum EE of 380 × 10^6^ MJ and 80 × 10^6^ MJ is observed in concrete and steel buildings, respectively (Fig. 10b). The increasing trend of EE in both columns and beams was almost the same. But, beams consume more EE as compared to columns as can be seen in Fig. 10a, b.

The differences in consumed embodied energy in steel rebar and concrete are not negligible in sustainable and more energy-efficient design. Gan et al.^51^ studied the EE of tall buildings with a comparison of concrete, steel, and composite structures. The results confirmed that the composite and concrete structures generated more emissions than the steel structure considering steel recovery. Li et al.^52^ compared concrete, light steel, and timber buildings, and the outcomes showed that the concrete structure consumed the highest emissions. However, multi-story building design frequently links the steel members with the RCC floor, and this exercise significantly raises the above differences. Increasing the building height/floor the greater the dissimilarities in the consumption of embodied energy (Fig. 10). For the 52-storey building, the embodied energy of the reinforcement building is 52% less than the EE of the RCC building (i.e., 650 MJ × 10^6^ vs. 1000 MJ × 10^6^). This means that reinforcement/steel structures consume less energy as compared to RCC buildings. Furthermore, the steel building type guarantees the more reasonable values of EE up to 40–56 stories; the RCC building type, up to 60–70 stories. Further than these heights, the steel (reinforcement), structure type becomes inefficient. In another way, for more than 20 floors, the core structural type and members (e.g., columns and beams) guarantee more economical values of the embodied energy (EE).

#### Effect of beam dimensions

**Figure 11:**
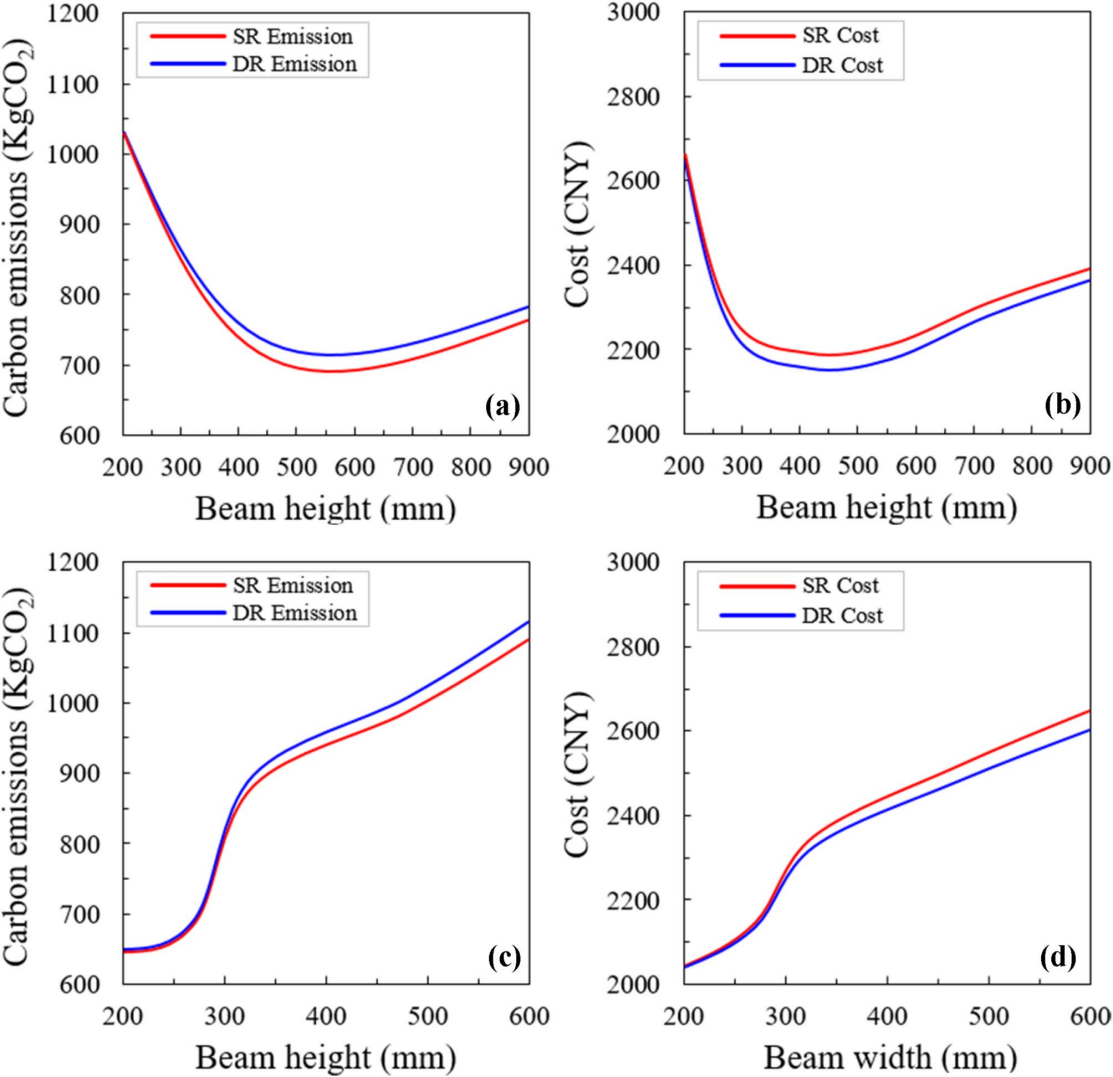
Optimized emissions and costs according to the changes in the sectional dimensions: (**a,b**) beam height; and (**c,d**) beam width.

The reliability of the proposed method is verified by optimization analysis. For this purpose, a comprehensive parametric study is conducted to examine the effect of different parameters on the sustainable design of RCC beams. First, the changes in beam height and width were examined, and the costs and optimized emissions according to the changes in beam size are shown in Fig. 11a, b. The results indicated that, by increasing the beam height, the optimized emissions and costs increased first and then decreased for both the singly reinforced (SR) and doubly reinforced (DR) beams, and a feasible range of 550–600 mm was observed. When the beam height was below this range, a SR section was preferred for the sustainable design, whereas, the situation was just the opposite when the height exceeded this range. Concerning the influences of beam width, the results in Fig. 11c, d imply that smaller sectional width can significantly benefit both the minimum emission and the minimum cost optimization.

#### Effect of column width

**Figure 12:**
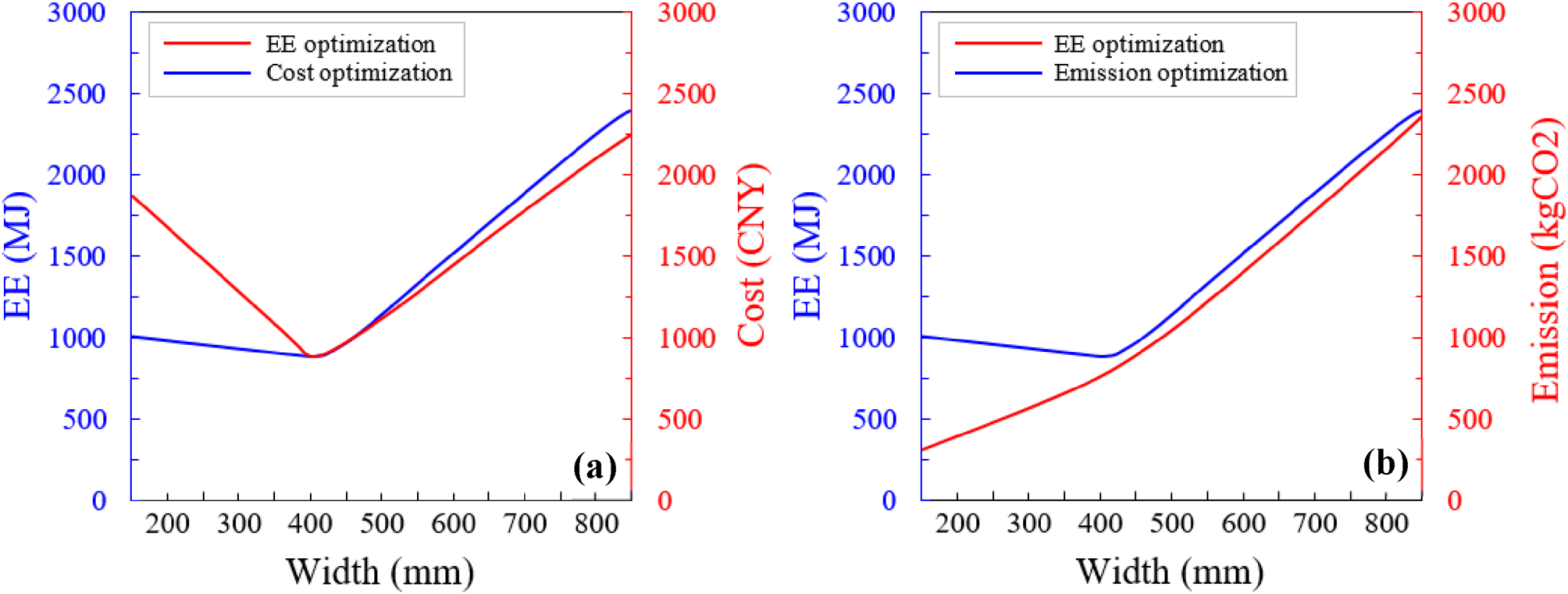
Objective functions vs. column section size obtained by sustainable design optimization. (**a**) Cost optimization and (**b**) CO_2_ emission optimization.

The optimization study of the sustainable building design was carried out using embodied energy (EE), CO_2_ emissions, total costs, and objective functions. The optimization results for the two best sections are presented in Fig 12a, b. In both figures, the calculated values of the objective function for EE are compared with the counterparts for the CO_2_ emissions and costs. The objective function values are proportionate to the dimension of the section when the section dimension is sufficient to require only the minimum steel bars. On the other hand, as the dimension of the section rises with a decrease of steel bars, the values object function acts contrarily. As can be observed from Fig. 12a, b, when the used amounts of concrete and steel bars are maximized and minimized, respectively, then the cost objective function has its lowest value. Furthermore, as the dimension of the section reduces, the carbon emissions objective function would slightly reduce due to its less sensitive behavior than steel bars. The EE objective function occurs between the CO_2_ emissions and costs function profiles. In addition, the optimized section of EE is close to the costs-optimized section in the compressive failure mode. This indicates that the tension-controlled section requires more steel bars to secure strength compared to the compression-controlled section.

#### Effect of eccentricity

**Figure 13:**
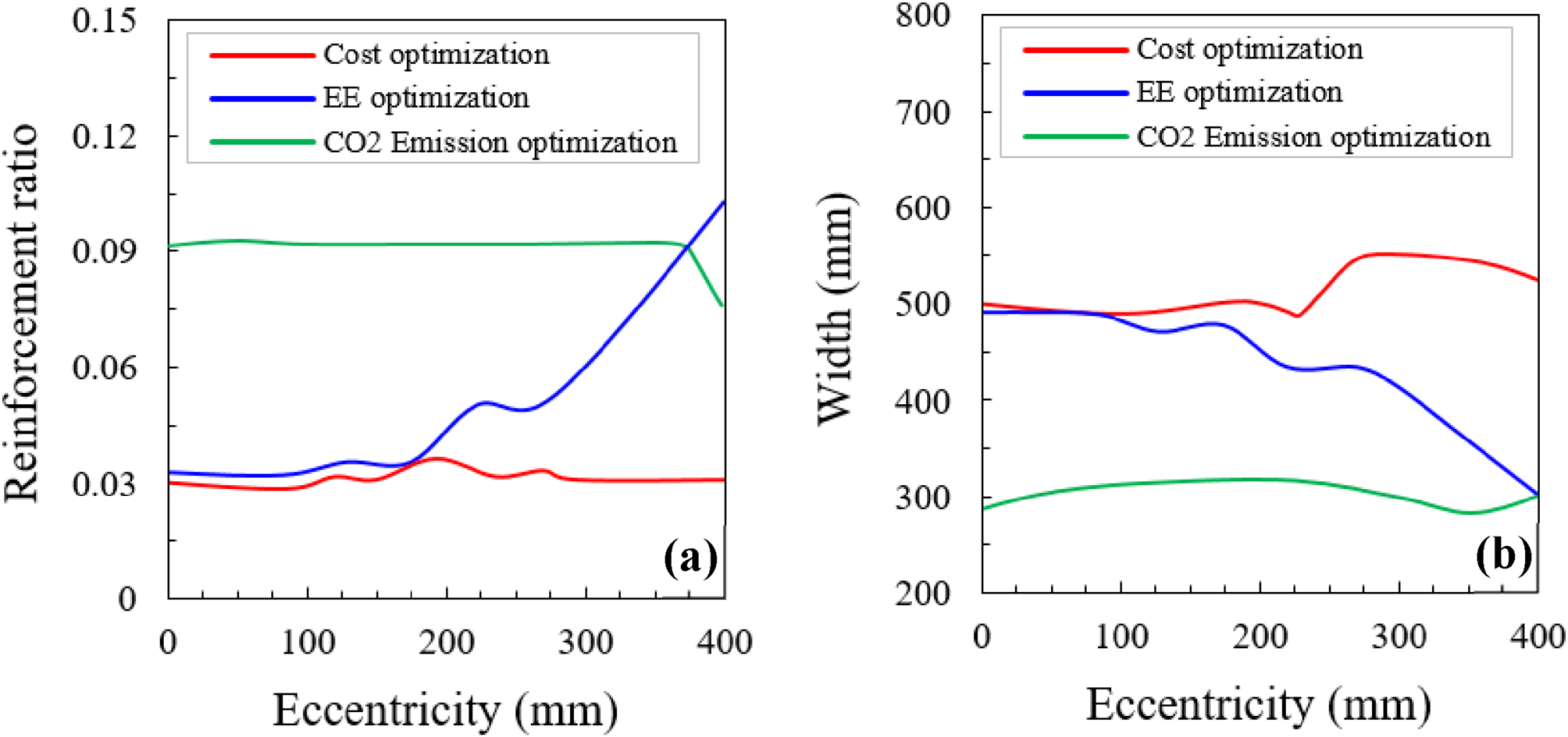
(**a**) Effect of reinforcement ratio on the cost optimization, CO_2_ emissions, and EE optimization concerning the eccentricity and (**b**) effect of column width on the cost optimization, CO_2_ emissions, and EE optimization concerning the eccentricity.

The variation in the ratio of the steel bars according to the eccentricity is shown in Fig. 13a. The ratios of steel bars are calculated for the optimized sections of cost, CO_2_ emissions, and embodied energy (EE). The CO_2_-optimized section retains the maximum steel ratio while the cost-optimized section preserves almost the minimum steel bar ratio (Fig. 13a). This indicates that the lower steel bar ratio is more beneficial for cost optimization, meanwhile the cost of the steel bar was higher than that of concrete. In contrast, the CO_2_ emissions optimization needs a high steel ratio due to the higher amount of CO_2_ released from concrete. On the other hand, the EE optimization retains a small steel proportion in the compressive failure characteristics whereas it incites the rise of steel ratio when the eccentricity changes into the tensile behavior. This is attributed to a comparatively higher influence of concrete on embodied energy optimization.

Figure 13b shows the relationship between the eccentricity and column width for three optimized sections. The most cost-effective section might be characterized via the least cross-sectional area with the minimum steel bars. Irrespective of the steel bars, the carbon emissions optimization produces the smallest cross-sectional area among the reasonable ones (Fig. 13b). The embodied energy optimization yields the minimum reinforcement ratio (Fig. 13b). But, it gives a large cross-sectional area as compared to cost-optimized section. Since the optimized sections differ according to the failure behavior, in cases of embodied energy and cost optimizations, much attention must be paid to the column design to determine the optimization with a proper safety margin.

#### Effect of material strength

**Figure 14:**
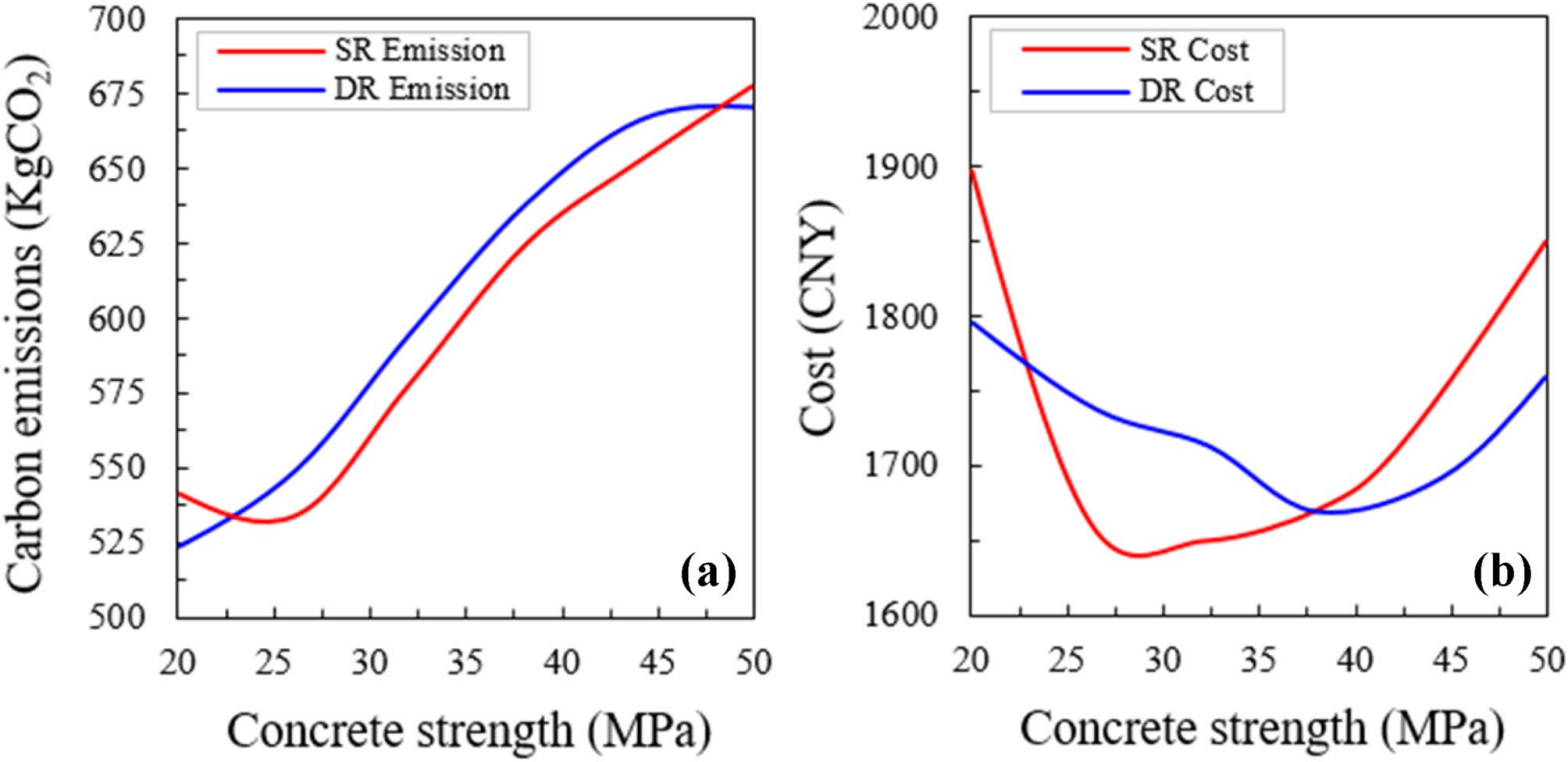
(**a**) Optimized emissions and (**b**) costs according to the changes in the concrete strength.

Figure 14 displays the results of optimized emissions and costs according to the changes in the strength of concrete. The optimal solutions that adopted 25 MPa and 30 MPa concrete can achieve the lowest emissions among all scenarios for the minimum emission optimization, respectively for both beams (Fig. 14a, b). However, higher concrete strength as 37 MPa and 44 MPa proved to be better for the minimum cost optimization. The minimum emission solution could diminish the emissions by around 15% compared with the minimum cost optimization at an added cost of 8%. Also, further study showed that high-strength concrete could be useful to decrease the beam size. The total emissions from concrete casting may be increased owing to the higher production emission coefficients.

#### Load effect

**Figure 15:**
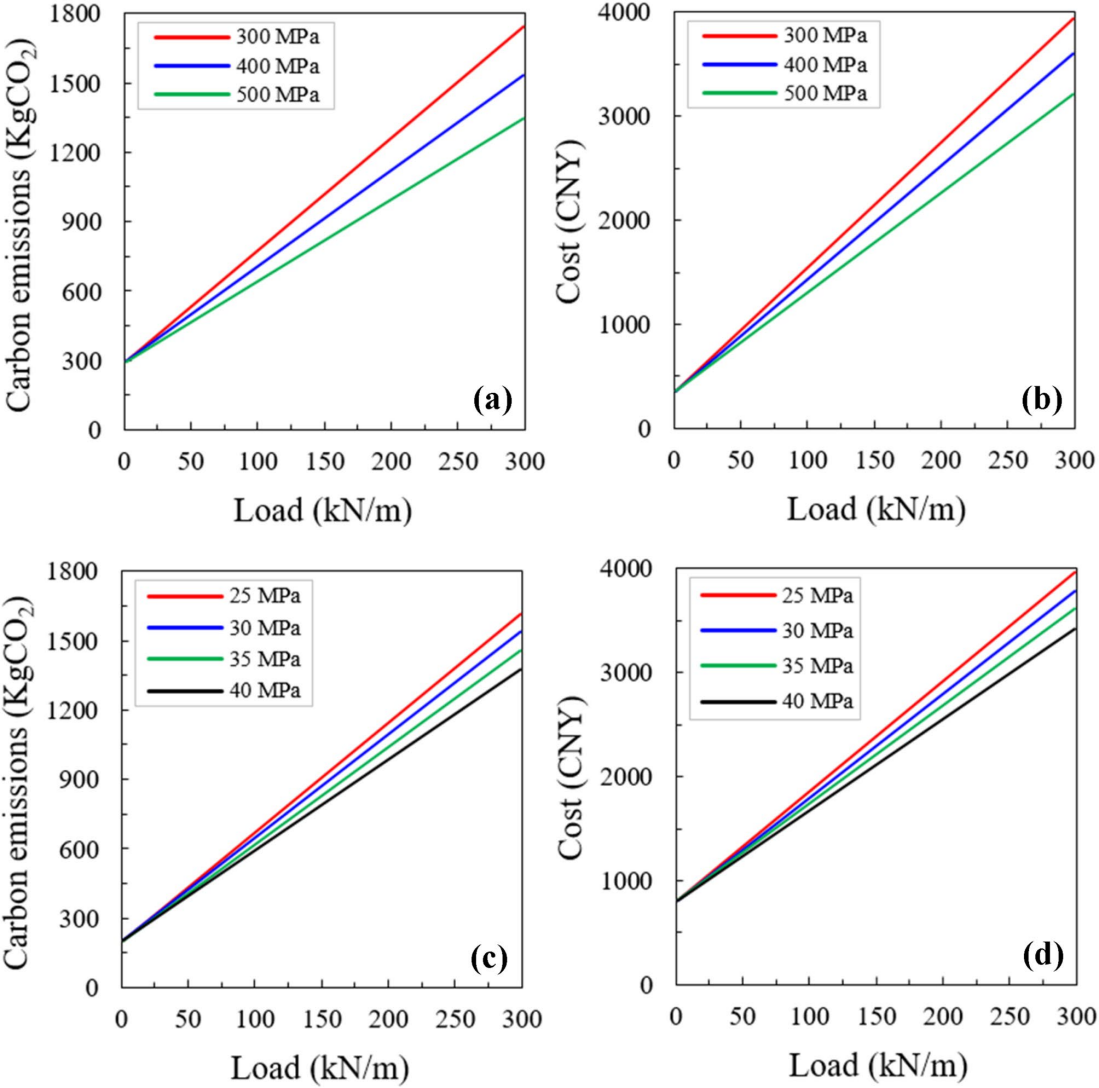
The minimum emission and cost optimization for the SR beam section, respectively as a function of the loads corresponding to (**a,b**) different main bar strengths; and (**c,d**) different concrete strengths.

In this study, it is assumed that the desired beam is subjected to a design load of 60 kN/m. A comprehensive study is conducted considering the cost optimization and minimum emission. The detailed outcomes of the singly reinforcement (SR) beam are demonstrated in Fig. 15. An overall rising trend was identified in the costs and optimized emissions by increasing the loads (Fig. 15). 500 MPa main bars proved to be the most effective for the minimum emission optimization, yet suitable strength for main bars varied from 300 to 500 MPa for the minimum cost optimization as shown in Fig. 15a, b. Fig. 15c, d exhibit that the viable concrete strength slowly rose related to the rise in the loads for cost optimization and minimum emission. Similar findings were also noted in the optimization of the doubly reinforced (DR) beam. So, the doubly reinforced beam was found to be more suitable than the SR section if the beam was subjected to relatively high loads.

**Figure 16:**
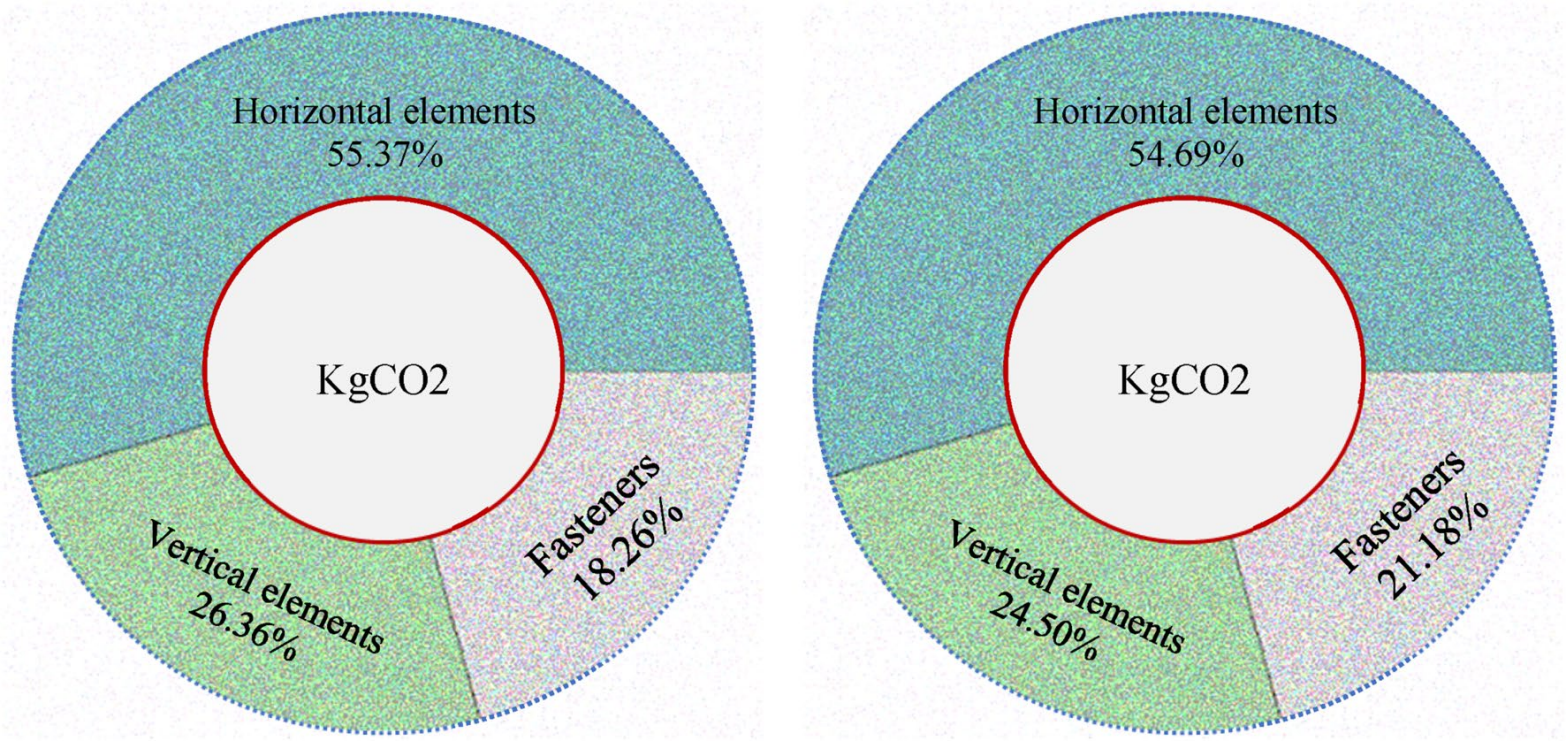
The contribution of different load-bearing elements in the superstructure to gas emissions, from^54^.

An investigation of the comparative contribution of structural elements and mechanical pins showed that their share ranges from 2.5 to 4.8% in the case of non-renewable energy, and from 2.8 to 3.8% in the case of carbon emissions (Fig. 16). The contribution of mechanical elements and structural components in this figure is marginally higher but consistent with the outcomes of Zhang and Zhang^34^ study. He also found that the connections and fasteners accounted for about 17% and 15% of the embodied effect respectively. Moreover, the carbon emission rate of steel connectors is approximate and provides an order of magnitude but is not exact^35,41^.

#### A cost–benefit analysis for the sustainable design

**Figure 17:**
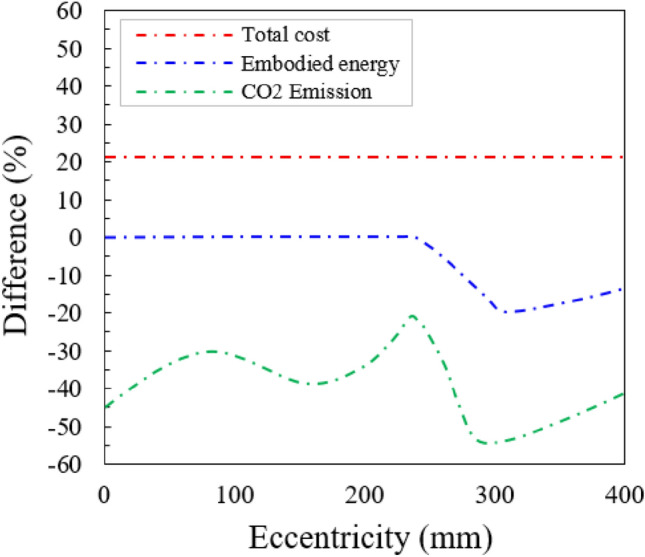
Cost–benefit analysis results for the embodied energy and CO_2_ emissions. For a 10% cost increase, the reductions in embodied energy and CO_2_ emissions are measured according to the eccentricity.

Based on the outcomes presented in the previous section, it is useful to increase the amount of steel bars to optimize the CO_2_ emissions and EE in reinforcement-concrete column design. However, the increase of steel bars may increase the cost, which might be inevitable in sustainable design. Under the assumption that a 10% cost increase is acceptable in the design practice. According to the variation in eccentricity, the curves of total costs, EE, and CO_2_ emissions are shown in Fig. 17. As shown in Fig. 17, the decreasing rate of the EE is very small when the value of eccentricity was 0 to 250 mm while the CO_2_ emissions might be significantly reduced by −21% to −55 %. These results highlight that the cost-optimized section increases faster than the carbon emissions-optimized section. On the other hand, as the eccentricity rises the diminishing rate of carbon emissions decreases as shown in Fig. 17. The carbon emissions and EE of the section remarkably decrease at the higher value of eccentricity (Fig. 17). This might be attributed to the extensive decrease in the cross-sectional area, involving the relatively smaller increase of steel bars. The plateaus of CO_2_ emissions and EE curves at the large eccentricities indicate that the effect of concrete may weaken after passing a certain magnitude of eccentricity. For that reason, it is very promising that sustainable design can effectively reduce the CO_2_ emissions and embodied energy of this mode.

Amer et al.^53^ demonstrated that sustainable and efficient energy-efficient design might reduce 10% of the EE in the reinforcement-concrete beam design by a 5% cost increase. This research also indicated that the carbon emissions might be reduced by more than 60% and EE might be decreased by more than 20% by sustainable design. As a result, it should be stressed that both carbon emissions and EE can be remarkably reduced with a small increase in cost in sustainable design for the RCC column.

## Conclusions

The current study established a method for the sustainable design of RCC multi-story building, which is a distinct optimization issue corresponding to the reduction of construction costs and embodied emissions. The following conclusions are drawn:

1. Optimizing the design of structural members for EE leads to embodied energy decreases of around 12% at the expense of an increase in cost of about 5% when compared to a member that has undergone cost optimization. The exact quantity of embodied energy reduction is reliant upon the steel bars to concrete cost ratio (*R*).
2. The minimum cost and emissions of all solutions were 551 kgCO_2_ and 1682 CNY (Chinese Yuan) for the doubly-reinforced beam, and 560 kgCO2 and 1688 CNY for the singly-reinforced beam. For the more sustainable design, the beam width was always 200 mm relevant to the total cost and minimum emissions. The singly reinforced beam was observed to be a sustainable option when the beam was exposed to a severe environment, and the doubly reinforced beam was preferred if the designed load was relatively high.
3. High strength steel bars can contribute to the minimum emission optimization. However, in the minimum cost optimization, the feasible concrete strength was usually higher for the sustainable design than the minimum emission optimization, and slowly rose as the loads increase.
4. The total costs of the RCC columns can be increased by increasing the amount of steel bars. In addition, the cost–benefit analysis showed that when increasing the cost by 10%, the embodied energy can be reduced by up to about 20% and the CO_2_ emissions can be diminished by up to around 60% in the RCC columns.

Future research involves the optimal design of reinforced concrete buildings for sustainability dynamic loads and wind loads in which the EE and cost objective functions are treated simultaneously. Additionally, CO_2_ emissions or greenhouse gas emissions will be considered as an alternative indicator for the sustainable design of multi-story RC and composite buildings.

## Data Availability

The data used to support of this study are included within the article.
